# FGF2-induced effects on transcriptome associated with regeneration competence in adult human fibroblasts

**DOI:** 10.1186/1471-2164-14-656

**Published:** 2013-09-26

**Authors:** Olga Kashpur, David LaPointe, Sakthikumar Ambady, Elizabeth F Ryder, Tanja Dominko

**Affiliations:** 1 Department of Biology and Biotechnology, Worcester Polytechnic Institute, 100 Institute Road, Worcester, MA 01609, USA; 2 Department of Cell and Developmental Biology, University of Massachusetts Medical School, 55 Lake Avenue North, Worcester, MA 01655, USA; 3 Department of Biomedical Engineering, Worcester Polytechnic Institute, 100 Institute Road, Worcester, MA 01609, USA

**Keywords:** Transcriptome, Human fibroblasts, Fibroblast growth factor (FGF2), Wound healing, Regeneration

## Abstract

**Background:**

Adult human fibroblasts grown in low oxygen and with FGF2 supplementation have the capacity to tip the healing outcome of skeletal muscle injury – by favoring regeneration response *in vivo* over scar formation. Here, we compare the transcriptomes of control adult human dermal fibroblasts and induced regeneration-competent (iRC) fibroblasts to identify transcriptional changes that may be related to their regeneration competence.

**Results:**

We identified a unique gene-expression profile that characterizes FGF2-induced iRC fibroblast phenotype. Significantly differentially expressed genes due to FGF2 treatment were identified and analyzed to determine overrepresented Gene Ontology terms. Genes belonging to extracellular matrix components, adhesion molecules, matrix remodelling, cytoskeleton, and cytokines were determined to be affected by FGF2 treatment.

**Conclusions:**

Transcriptome analysis comparing control adult human fibroblasts with FGF2-treated fibroblasts identified functional groups of genes that reflect transcriptional changes potentially contributing to their regeneration competence. This comparative transcriptome analysis should contribute new insights into genes that characterize cells with greater regenerative potential.

## Background

During development, distinct cell phenotype differentiation is guided by finely tuned and orchestrated changes in transcriptional activity of specific groups of genes that become gradually activated (lineage-specific), gradually repressed (stem cell and progenitor cell genes), or whose activity does not change substantially (housekeeping genes). Ultimately, analyzing the transcriptome of a cell type offers an opportunity to broadly identify transcripts that define it. In addition to these either developmentally regulated or artificially induced phenotype changes that are accompanied by distinct transcriptional changes, a transcriptome of any given cell type can vary substantially depending on cell cycle [1-3], passage number, and environmental factors such as oxygen concentration [4], temperature, and presence of serum [5].

Another important factor that causes transcriptional changes and is crucial for maintaining a cell phenotype is growth substrate. For example, maintenance of undifferentiated state of embryonic stem cells is dependent on favorable substrate, composed of laminin [6-9], vitronectin [9-12], fibronectin [9], and collagen IV [8]. In addition to chemical composition, physical properties of substrate also determine cell fate. Roughness and stiffness of the surfaces have also been shown to affect developmental plasticity of cells. Smooth and rigid glass surface supports undifferentiated phenotype, while rough and soft substrates promote differentiation [13,14].

Lastly, presence of various growth factors in culture media can have a significant effect on a cell transcriptional activity and consequently its phenotype. FGF2 is a mesenchyme-derived growth factor that displays mitogenic, migratory, and morphogenic functions and is also known to play role in angiogenesis, organ development, organ regeneration, and wound healing [15]. Contrary to its predominantly mitogenic effects on differentiated cell types, FGF2 is absolutely required for maintenance of expression of stemness-related genes. With respect to wound healing, FGF2 has been studied as a potential therapeutic anti-scarring agent [15-17].

We have previously investigated the effects of the aforementioned important cell culture conditions, FGF2 and culture surface, on adult human fibroblasts. We have observed that adult human fibroblasts demonstrated FGF2- and surface-mediated induction of some endogenous stem cell genes and a capacity to acquire a more developmentally plastic phenotype. This low level of activation of stem cell genes was not sufficient for induction of a phenotypic conversion into a pluripotent cell phenotype [18]. However, when transplanted into skeletal muscle injury, adult human fibroblasts grown in low oxygen and with supplementation of FGF2 had the capacity to tip the healing outcome of skeletal muscle injury – by favoring regeneration response *in vivo* over scar formation [19]. The wound repair process consists of several phases, including immediate response to injury, inflammatory response, cell proliferation and migration, ECM contraction, and ECM remodeling. The roles of dermal fibroblasts in wound healing have been described [20] and in mammals fibroblasts facilitate collagen deposition and formation of a scar. The cascade of molecular events leading to scar formation involves increased proliferation and migration of fibroblasts in response to growth factors [21], production and organization of specific ECM components [22,23], and acquisition of an actin-dependent contractile phenotype [24]. The wound repair process is complete by formation of a scar (disorganized extracellular matrix, mainly collagen) [20].

In this study, we compared transcriptomes of control fibroblasts and regeneration- competent fibroblasts to determine whether transcriptional profile that characterizes regeneration-competent cells reflects disregulation of genes involved in the default wound healing pathway leading to scar formation – turning the cells into a more pro-regenerative phenotype.

## Results

### The effect of cell growth surface and FGF2 on fibroblast transcriptome

To obtain a sense of the effects of surface and FGF2 treatment on global transcription, two independent samples (in three technical replicates each) of human dermal fibroblasts grown on glass, glass with FGF2, plastic, and plastic with FGF2 were hybridized to the Human Whole Genome OneArray® microarray, which contains 29,187 human oligonucleotide probes. Background-corrected intensity data was normalized and filtered, which identified 11,124 probes of detectable level of intensity (Additional file 1). The gene expression dataset is of excellent quality as indicated by Pearson’s correlation coefficients for biological replicates: 0.987 for glass, 0.973 for glass with FGF2, 0.960 for plastic, and 0.971 for plastic with FGF2 (Additional file 2). To investigate cell culture effects, we examined significantly differentially expressed gene probes using moderated t-statistic and based on the false discovery rate (FDR) cutoff value of 0.05. Comparison of transcriptomes between cells grown on glass and plastic in the absence of FGF2 did not identify any differentially expressed genes. However, FGF2-induced changes in gene expression depended on surface.

**Figure 1:**
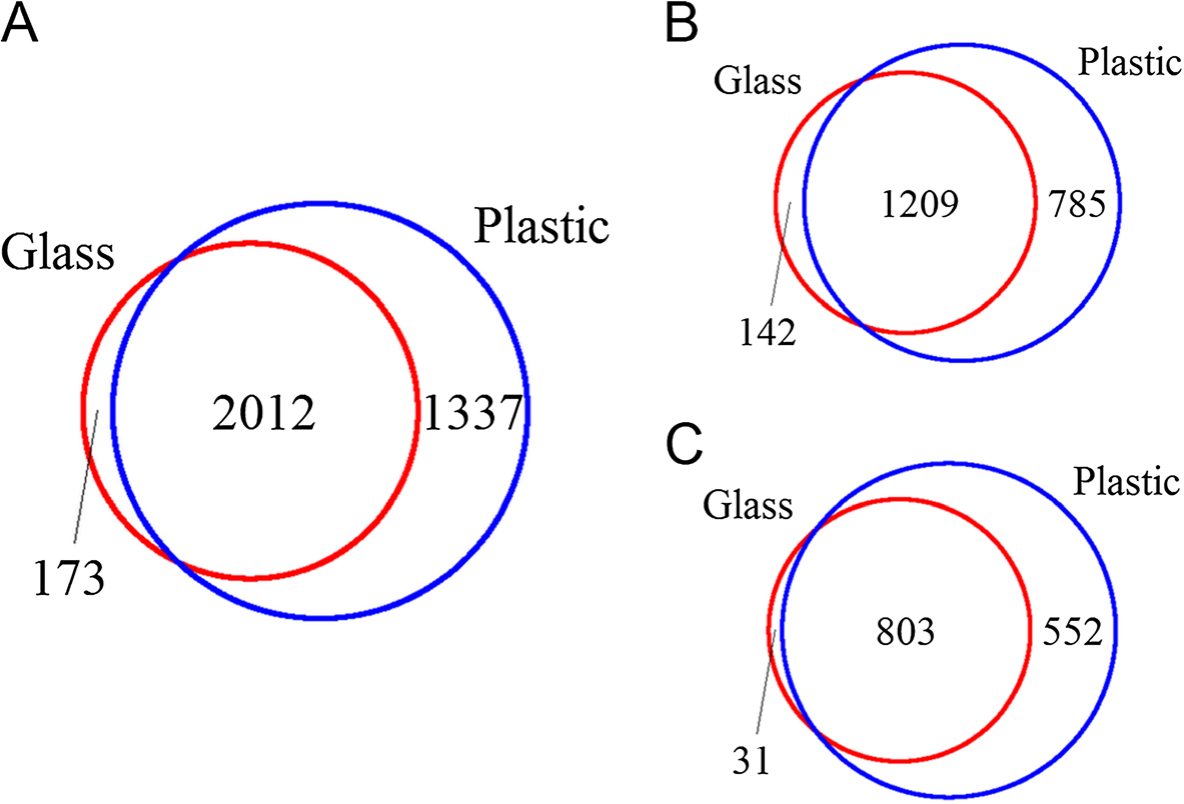
**FGF2 changes gene expression in human fibroblasts. A**. Venn diagram showing the overlap between differentially expressed gene probes on plastic and glass. **B**. Venn diagram depicting the overlap between upregulated gene probes on plastic and glass. **C**. Venn diagram depicting the overlap between downregulated gene probes on plastic and glass.

**Figure 2:**
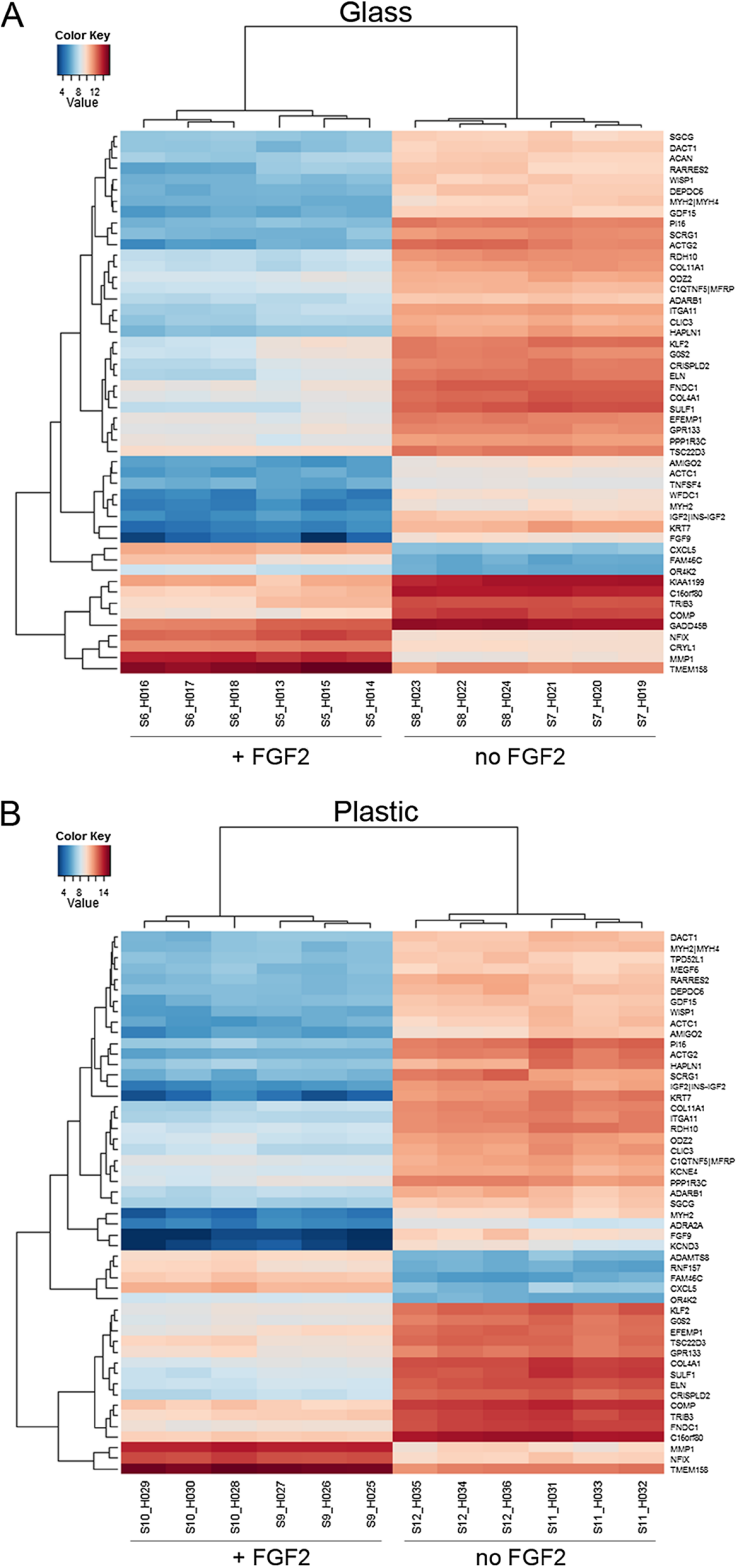
**Top 50 differentially expressed genes due to FGF2 treatment. A**. Heat map showing level of gene expression on glass. **B**. Heat map showing level of gene expression on plastic.

FGF2 had a more prominent effect on cells when grown on plastic than on glass, as determined by the overall increased number of differentially expressed gene probes (3,349 on plastic versus 2,185 on glass) (Figure 1A). In response to FGF2 treatment, 2,012 differentially expressed gene probes (1,767 genes) were identified that were disregulated on both surfaces: 1,209 common gene probes were upregulated (1,071 genes) (Figure 1B) and 803 common gene probes downregulated (696 genes) (Figure 1C). In addition to these common genes, FGF2 treatment disregulated 173 unique gene probes (168 genes: 139 upregulated and 29 downregulated) on glass and 1,337 unique gene probes (1,282 genes: 753 upregulated and 529 downregulated) on plastic (Figure 1). The complete list of differentially expressed gene probes on glass and on plastic can be found in Additional file 3 and Additional file 4, respectively. The top 50 significantly differentially expressed genes are represented in the heat maps (Figure 2A and B, respectively). All further analyses were performed on genes whose expression was disregulated in cells grown in the presence of FGF2 on plastic.

### Gene ontology analysis

**Figure 3:**
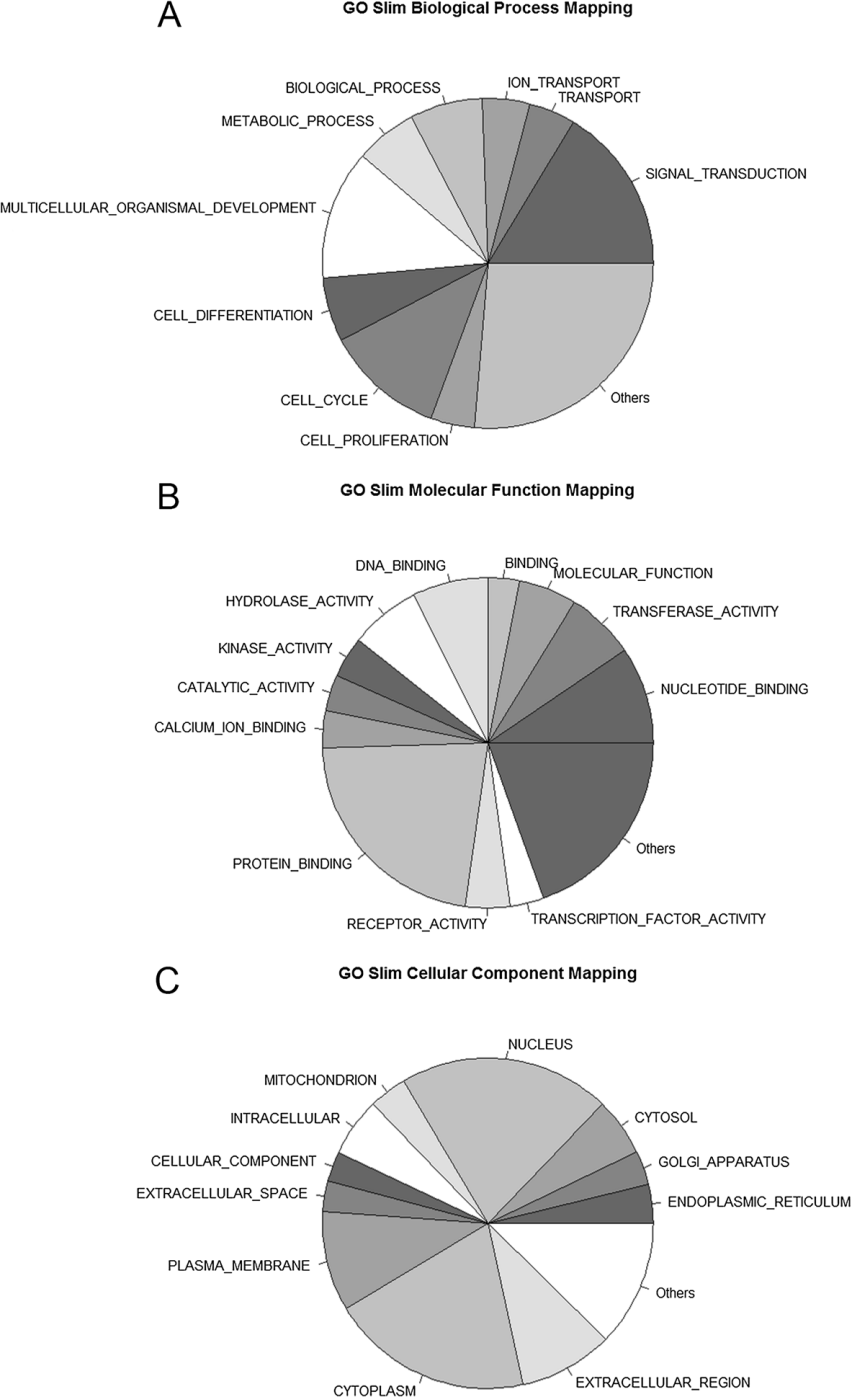
**GO terms characterizing FGF-affected genes. A**. Terms associated with biological process. **B**. Terms associated with molecular function. **C**. Terms associated with cellular component.

Differentially expressed genes were analyzed for functional enrichment. To determine the functions of the genes affected by FGF2 treatment and consequently identify the cellular processes that are affected by these transcriptional changes, we performed Gene Ontology (GO) analysis. First, all significantly differentially expressed genes were analyzed to determine broad GO term overrepresentation using GO slim analysis. GO slim analysis identified broad terms describing biological processes (Figure 3A), molecular functions (Figure 3B), as well as cellular components to which they belong (Figure 3C). Additional file 5 includes the number of gene probes representing each GO slim term. A total of 664 overrepresented GO terms (p < 0.05) associated with biological processes were identified. These included genes involved in regulation of cell cycle, cardiovascular system development, extracellular matrix organization, cell proliferation, cell adhesion, regulation of angiogenesis, cell migration, and wound healing. Seventy seven overrepresented GO terms (p < 0.05) were associated with molecular function. The genes belonged to extracellular matrix structural constituents, genes regulating collagen, heparin, integrin binding, and genes regulating cytokine activity. Sixty five overrepresented GO terms (p < 0.05) were associated with cellular components and belonged primarily to extracellular components (Additional file 6).

### Expression of genes associated with wound healing

As FGF2-treated human dermal fibroblasts were previously shown to participate in wound healing of volumetric skeletal muscle by contributing directly to the pool of satellite PAX7 positive cells and by stimulating regeneration of endogenous skeletal muscle tissue [19], we focused further analysis of differentially expressed genes to those that play a role in wound healing and could be uniquely identifying regeneration-competent fibroblasts.

Overall, select genes belonging to extracellular matrix and its remodeling, inflammation, cytoskeleton and migration, and growth factor signaling were found to be affected by FGF2.

#### Extracellular matrix, matrix remodeling enzymes, and adhesion molecules

**Figure 4:**
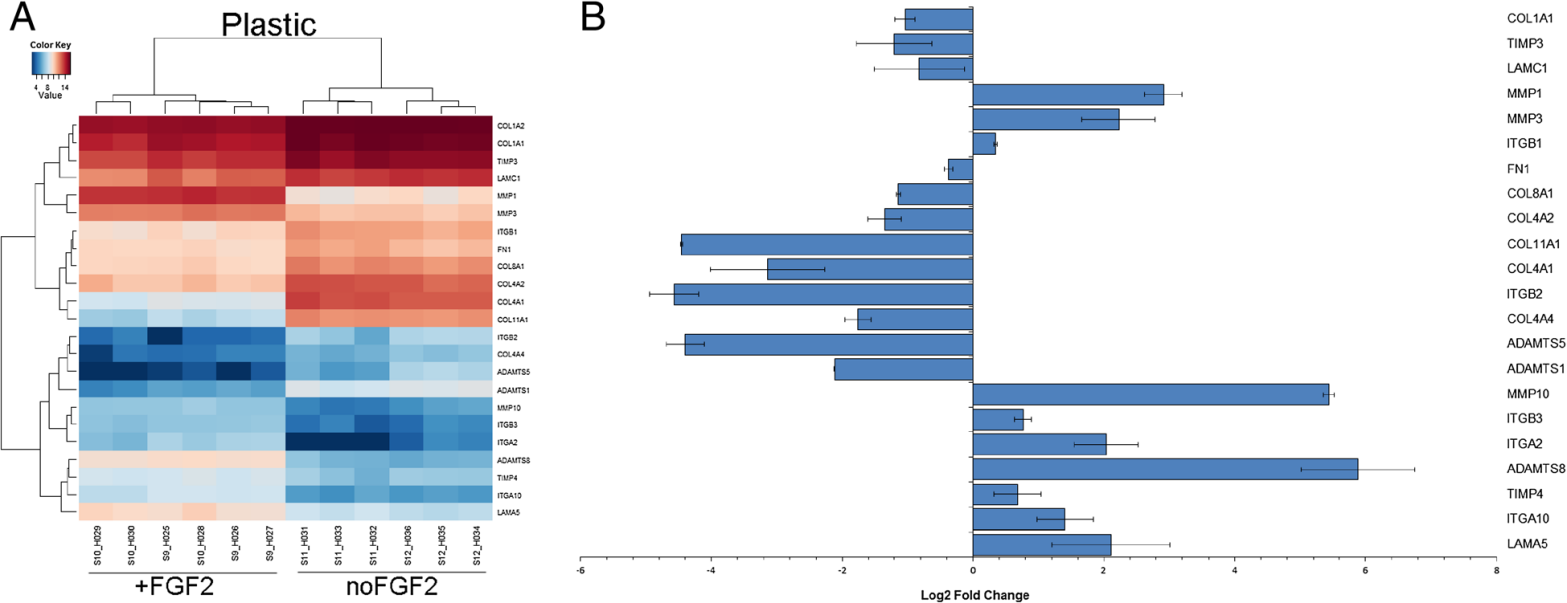
**FGF2 affects expression levels of genes associated with extracellular matrix remodelling. A**. Heat map showing expression levels of select genes as identified by microarray analysis. **B**. qRT-PCR validation of microarray data for select genes. Expression levels were normalized to ACTB and are represented as log2 Fold Change (FGF2-treated compared to untreated). Error bars represent SEM. qRT-PCR for COL1A2 did not show change in the expression levels.

FGF2 treatment led to downregulation of most collagens (COL11A1, COL4A2, COL8A1, COL5A1, COL1A1, COL12A1, COL15A1) and fibronectin (FN1) and to upregulation of several laminins (LAMB1, LAMB3, LAMA3). FGF2 increased expression of select metallopeptidases (stromelysines MMP3, MMP10, and MMP11; MMP1; ADAMTS8), and metallopeptidase inhibitor TIMP4. Among downregulated genes were TIMP3, and several other ADAMTS proteinases (ADAMTS5 and ADAMTS1). Different members of integrin family responded by significant upregulation (ITGA2, ITGA10, ITGB3) or downregulation (ITGA11, ITGB2). Significantly disregulated genes identified by the microarray are presented in Table 1. Expression levels of select target genes identified by the microarray (Figure 4A) were examined by qRT-PCR (Figure 4B).

**Table 1:** ECM, adhesion, and matrix remodeling genes affected by FGF2 treatment

**Symbol**	**Name**	**Log2 fold change**	**Fold change**	**Adjusted p-value**
**ECM**				
**Collagens**				
COL21A1	Collagen, type XXI, alpha 1	2.99	7.97	4.40E-06
COL14A1	Collagen, type XIV, alpha 1	2.25	4.74	3.58E-05
COL13A1	Collagen, type XIII, alpha 1	1.62	3.07	0.003150228
COL18A1	Collagen, type XVIII, alpha 1	1.40	2.64	0.026150843
COL6A3	Collagen, type VI, alpha 3	1.02	2.02	0.026527481
COL10A1	Collagen, type X, alpha 1	0.61	1.52	0.033185205
COL27A1	Collagen, type XXVII, alpha 1	−0.59	−1.50	0.000481652
COL16A1	Collagen, type XVI, alpha 1	−0.86	−1.82	0.000501675
COL1A2	Collagen, type I, alpha 2	−0.92	−1.90	0.001062514
COL12A1	Collagen, type XII, alpha 1	−1.16	−2.23	0.001285122
COL5A1	Collagen, type V, alpha 1	−1.17	−2.25	0.000370081
COL1A1	Collagen, type I, alpha 1	−1.26	−2.40	2.50E-05
COL15A1	Collagen, type XV, alpha 1	−1.40	−2.64	6.04E-06
COL8A1	Collagen, type VIII, alpha 1	−1.92	−3.77	5.23E-08
COL5A2	Collagen, type V, alpha 2	−1.99	−3.96	0.000570978
COL5A3	Collagen, type V, alpha 3	−2.24	−4.72	8.97E-06
COL4A4	Collagen, type IV, alpha 4	−2.34	−5.07	0.001283892
COL4A2	Collagen, type IV, alpha 2	−2.40	−5.28	3.02E-14
COL11A1	Collagen, type XI, alpha 1	−4.27	−19.37	4.50E-12
COL4A1	Collagen, type IV, alpha 1	−4.61	−24.43	3.58E-05
**Laminins**				
LAMA5	Laminin, alpha 5	1.74	3.35	5.24E-06
LAMB1	Laminin, beta 1	0.68	1.60	0.026626565
LAMA4	Laminin, alpha 4	0.66	1.58	0.030288975
LAMA3	Laminin, alpha 3	0.62	1.54	0.033413419
LAMC2	Laminin, gamma 2	−0.78	−1.72	0.001416011
LAMA2	Laminin, alpha 2	−0.81	−1.76	0.048073235
LAMB2	Laminin, beta 2 (laminin S)	−0.88	−1.84	0.016068977
LAMC1	Laminin, gamma 1 (formerly LAMB2)	−1.34	−2.53	8.56E-05
**Fibronectins**				
FNDC4	Fibronectin type III domain containing 4	1.27	2.41	0.000256057
FNDC3A	Fibronectin type III domain containing 3A	0.81	1.75	0.036705362
FNDC3B	Fibronectin type III domain containing 3B	−1.12	−2.17	0.001723239
FN1	Fibronectin 1	−1.14	−2.20	0.00019286
FNDC1	Fibronectin type III domain containing 1	−4.06	−16.64	2.29E-12
**Adhesion molecules**				
**Integrins**				
ITGA2	Integrin, alpha 2 (CD49B, alpha 2 subunit of VLA-2 receptor)	3.69	12.92	4.74E-06
ITGA10	Integrin, alpha 10	2.62	6.14	3.90E-07
ITGB3	Integrin, beta 3 (platelet glycoprotein IIIa, antigen CD61)	2.37	5.16	8.55E-05
ITGB1	Integrin, beta 1 (fibronectin receptor, beta polypeptide, antigen CD29 includes MDF2, MSK12)	−1.66	−3.15	6.42E-06
ITGBL1	Integrin, beta-like 1 (with EGF-like repeat domains)	−2.31	−4.97	6.78E-08
ITGB2	Integrin, beta 2 (complement component 3 receptor 3 and 4 subunit)	−3.34	−10.15	0.000457236
**Cadherins**				
CDHR3	Cadherin-related family member 3	1.22	2.33	0.008261441
PCDHGC3	Protocadherin gamma subfamily C, 3	1.21	2.32	0.007298787
PCDH9	Protocadherin 9	1.19	2.29	0.001804197
PCDH10	Protocadherin 10	1.08	2.11	0.00089387
CDH11	Cadherin 11, type 2, OB-cadherin (osteoblast)	−0.72	−1.64	0.025796984
PCDHB2	Protocadherin beta 2	−0.76	−1.69	0.010987526
CDH2	Cadherin 2, type 1, N-cadherin (neuronal)	−2.05	−4.14	1.11E-05
PCDH7	Protocadherin 7	−2.36	−5.12	0.002357001
**Matrix remodeling**				
MMP1	Matrix metallopeptidase 1 (interstitial collagenase)	4.37	20.61	8.28E-12
ADAMTS8	ADAM metallopeptidase with thrombospondin type 1 motif, 8	3.31	9.94	1.65E-10
MMP27	Matrix metallopeptidase 27	1.90	3.72	4.70E-06
MMP10	Matrix metallopeptidase 10 (stromelysin 2)	1.81	3.52	0.000118221
MMP3	Matrix metallopeptidase 3 (stromelysin 1, progelatinase)	1.81	3.51	7.05E-06
TIMP4	TIMP metallopeptidase inhibitor 4	1.53	2.88	0.000259942
ADAM15	ADAM metallopeptidase domain 15	1.04	2.06	0.000703266
ADAMTSL4	ADAMTS-like 4	0.83	1.77	0.040463485
MMP11	Matrix metallopeptidase 11 (stromelysin 3)	0.82	1.76	0.009142377
ADAMTSL1	ADAMTS-like 1	0.68	1.60	0.024404628
THBS2	Thrombospondin 2	−0.60	−1.52	0.033809806
ADAM19	ADAM metallopeptidase domain 19	−0.82	−1.77	0.016522395
TIMP3	TIMP metallopeptidase inhibitor 3	−1.48	−2.78	8.49E-06
ADAM12	ADAM metallopeptidase domain 12	−2.72	−2.23	1.30E-08
ADAMTS1	ADAM metallopeptidase with thrombospondin type 1 motif, 1	−3.06	−8.32	4.12E-08
ADAMTS5	ADAM metallopeptidase with thrombospondin type 1 motif, 5	−3.99	−15.84	0.000108412

#### Cytoskeleton

**Figure 5:**
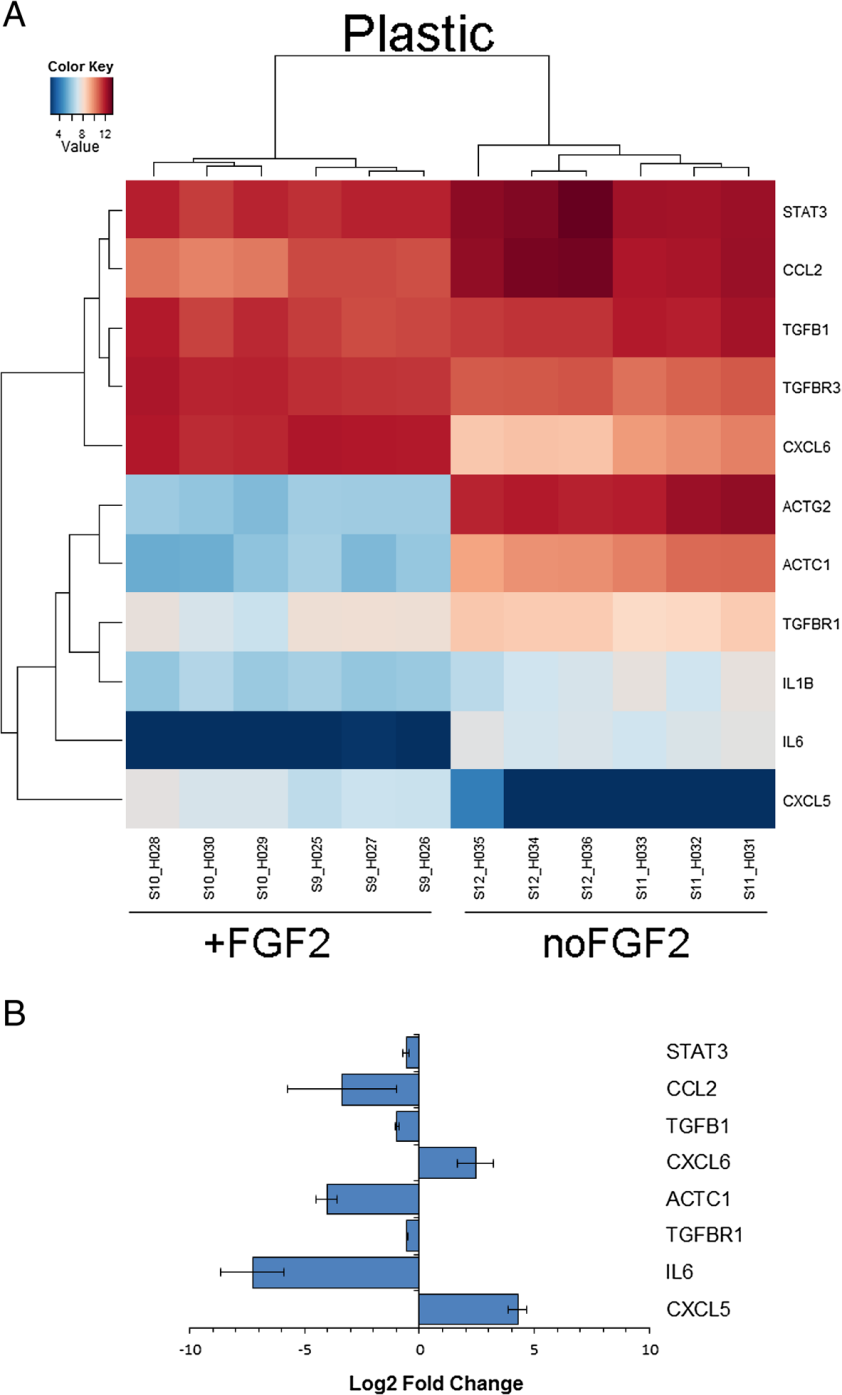
**FGF2 affects expression levels of cytoskeleton genes and chemokines. A**. Heat map showing expression levels of select genes as identified by microarray analysis. **B**. qRT-PCR validation of microarray data for select genes. Expression levels were normalized to ACTB and are represented as log2 Fold change (FGF2-treated compared to untreated). Error bars represent SEM. qRT-PCR for IL1B, ACTG2, and TGBR3 did not show change in the expression levels.

Another group of genes found to be regulated by FGF2 treatment were components of the cytoskeleton that are also involved in wound healing (Table 2). The most significant effect was observed on ACTC1 and ACTG2. Expression levels of ACTC1 and ACTG2 identified by the microarray (Figure 5A) were examined by qRT-PCR (Figure 5B).

**Table 2:** Cytoskeleton genes regulated by FGF2 treatment

**Symbol**	**Name**	**Log2 fold change**	**Fold change**	**Adjusted p-value**
TUBA4A	Tubulin, alpha 4a	1.29	2.44	0.000256991
TUBB3	Tubulin, beta 3 class III	1.14	2.20	0.00119515
TUBA1C	Tubulin, alpha 1c	0.95	1.93	0.002708545
TUBB2C	Tubulin, beta 2c	0.60	1.52	0.020192536
ACTA2	Actin, alpha 2, smooth muscle, aorta	−0.86	−1.81	0.00445901
ACTN1	Actinin	−1.38	−2.60	1.90E-05
TUBE1	Tubulin, epsilon 1	−1.42	−2.68	0.000182139
TUBG2	Tubulin, gamma 2	−2.71	−6.53	0.000159057
ACTC1	Actin, alpha, cardiac muscle 1	−4.38	−20.83	4.46E-11
ACTG2	Actin, gamma 2, smooth muscle, enteric	−6.01	−64.33	1.52E-13

#### Cytokines, their receptors, and downstream signaling molecules

Cytokines that were identified to be differentially expressed are listed in Table 3. FGF2-induced transcriptional increase was observed in genes associated with inflammation (CXCL1, CXCL5, PTGS2), and growth factor signaling (EGFR, HGF, MAPK1). Expression of pro-inflammatory cytokines interleukin-1B (IL1B) and IL6 decreased upon FGF2 treatment. Signal transducer and activator of transcription 3 (STAT3), which is a known downstream target of IL6 signaling, was downregulated as well as was another downstream IL6/STAT3 gene, CC chemokine ligand CCL2. Expression levels of all these targets identified by the microarray (Figure 5A) were confirmed by qRT-PCR (Figure 5B). FGF2 effect on expression of TGFB pathway genes included increase in TGFBR3 expression, decrease in TGFBR1, and decrease in TGFBI (Table 3). TGFB1 and TGFB3 were not significantly differentially expressed due to FGF2 treatment. qRT-PCR results for TGFB1 and TGFBR1 are presented in Figure 5B.

**Table 3:** Representative cytokines regulated by FGF2

**Symbol**	**Name**	**Log2 fold change**	**Fold change**	**Adjusted p-value**
**Chemokines**				
CXCL5	Chemokine (C-X-C motif) ligand 5	4.58	16.78	9.18E-07
CXCL6	Chemokine (C-X-C motif) ligand 6 (granulocyte chemotactic protein 2)	2.50	5.64	1.23E-07
CXCL1	Chemokine (C-X-C motif) ligand 1 (melanoma growth stimulating activity, alpha)	2.06	4.17	1.59E-05
CCL22	Chemokine (C-C motif) ligand 22	1.55	2.93	0.033547076
C5	Complement component 5	0.88	1.85	0.001196477
CCL26	Chemokine (C-C motif) ligand 26	−0.67	−1.59	0.027966918
CCL14	Chemokine (C-C motif) ligand 14	−1.15	−2.22	0.000191918
CCL25	Chemokine (C-C motif) ligand 25	−1.23	−2.35	0.007193543
CCL2	Chemokine (C-C motif) ligand 2	−1.79	−3.46	6.20E-06
**Chemokine receptors**				
CCRL1	Chemokine (C-C motif) receptor-like 1	2.02	4.06	1.37E-05
CCR10	Chemokine (C-C motif) receptor 10	0.98	1.97	0.043411565
CCR8	Chemokine (C-C motif) receptor 8	−0.58	−1.49	0.047496414
CXCR7	Chemokine (C-X-C motif) receptor 7	−2.26	−4.79	1.24E-06
**Interleukins**				
IL8	Interleukin 8	1.47	2.77	0.013242716
IL17D	Interleukin 17D	1.41	2.66	0.000124442
IL1RN	Interleukin 1 receptor antagonist	1.18	2.27	0.007602993
IL1B	Interleukin 1, beta	−1.10	−2.14	0.002340469
IL2	Interleukin 2	−1.19	−2.28	0.034673096
IL32	Interleukin 32	−1.19	−2.28	0.000974257
IL1RAP	Interleukin 1 receptor accessory protein	−1.91	−3.76	8.85E-06
IL33	Interleukin 33	−2.59	−6.02	0.000613327
IL6	Interleukin 6 (interferon, beta 2)	−5.07	−33.59	2.75E-07
**Interleukin receptors**				
IL17RD	Interleukin 17 receptor D	2.24	4.72	0.00026435
IL13RA2	Interleukin 13 receptor, alpha 2	1.84	3.58	3.65E-05
IL15RA	Interleukin 15 receptor, alpha	0.71	1.64	0.015033555
IL21R	Interleukin 21 receptor	−0.86	−1.82	0.004974339
IL20RB	Interleukin 20 receptor beta	−1.29	−2.45	0.000238276
IL1RL1	Interleukin 1 receptor-like 1	−1.99	−3.97	0.002708422
**STAT**				
STAT4	Signal transducer and activator of transcription 4	−1.51	−2.85	1.15E-06
STAT1	Signal transducer and activator of transcription 1	−0.87	−1.83	0.006745
STAT3	Signal transducer and activator of transcription 3 (acute-phase response factor)	−0.87	−1.83	0.021783
**Tumor necrosis factor family**				
TNFAIP8L1	Tumor necrosis factor, alpha-induced protein 8-like 1	3.90	14.93	8.62E-07
TNFRSF25	Tumor necrosis factor receptor superfamily, member 25	1.89	3.71	0.000432746
TNFSF10	Tumor necrosis factor (ligand) superfamily, member 10	1.74	3.34	0.005536998
TNFAIP8L3	Tumor necrosis factor, alpha-induced protein 8-like 3	1.31	2.48	9.01E-05
TNFRSF1B	Tumor necrosis factor receptor superfamily, member 1B	1.20	2.30	0.027881915
TNFRSF21	Tumor necrosis factor receptor superfamily, member 21	0.67	1.59	0.029823686
C1QTNF6	C1q and tumor necrosis factor related protein 6	−0.70	−1.62	0.035969462
TNFRSF10B	Tumor necrosis factor receptor superfamily, member 10b	−0.75	−1.68	0.025993715
C1QTNF3	C1q and tumor necrosis factor related protein 3	−0.81	−1.75	0.013142151
TNFAIP6	Tumor necrosis factor, alpha-induced protein 6	−0.85	−1.80	0.021930255
TNFAIP1	Tumor necrosis factor, alpha-induced protein 1 (endothelial)	−0.87	−1.83	0.002670969
TNFRSF10D	Tumor necrosis factor receptor superfamily, member 10d, decoy with truncated death domain	−1.14	−2.20	0.003564267
TNFRSF11B	Tumor necrosis factor receptor superfamily, member 11b	−1.59	−3.01	4.04E-05
C1QTNF5|MFRP	Membrane frizzled-related protein, C1q and tumor necrosis factor related protein 5 transcription unit	−2.42	−5.35	5.17E-10
TNFSF4	Tumor necrosis factor (ligand) superfamily, member 4	−2.83	−7.11	4.13E-09
**TGFΒ pathway**				
TGFBR3	Transforming growth factor, beta receptor 3	0.94	1.92	0.001259518
TGFBI	Transforming growth factor, beta-induced, 68kDa	−0.66	−1.58	0.014441976
TGFBR1	Transforming growth factor, beta receptor 1	−0.96	−1.91	0.005746781

## Discussion

The comparative transcriptome analysis described here demonstrates a unique molecular signature for induced regeneration-competent (iRC) fibroblasts compared with control fibroblasts. Consistent with the notion that these two cell types are distinctly different, we have used both cell types in *in vivo* regeneration experiments and demonstrated that the induced regeneration-competent fibroblasts participate in regenerative response of skeletal muscle (concomitant with decreased scar formation), contribute to the pool of newly established satellite cells (PAX7^+^ cells) in a mouse injury model, as well as form mature myotubes [19].

Identification of significantly differentially expressed genes and subsequent Gene Ontology analysis determined that a large number of genes important for the outcome of wound healing such as extracellular matrix genes, adhesion molecules, matrix remodeling genes, and genes involved in inflammation were regulated by FGF2 (Figure 3).

During dermal wound healing, fibroblasts are responsible for ECM production [17] and, here, we show that FGF2 treatment affects a number of genes involved in production and remodeling of ECM. FGF2 caused downregulation of a number of collagens such as collagen IV, collagen XI, collagen V, and collagen I, as well as caused upregulation of collagen XXI and collagen XIV (Table 1). qRT-PCR analysis confirmed downregulation of COL1A1, COL4A1, COL4A2, COL4A4, COL8A1, and COL11A1 (Figure 4B). FGF2 was previously shown to downregulate expression of interstitial collagen I and III [16]. Collagen I is a major component of ECM in skin, and during wound healing is the main scar forming collagen. Collagen IV is a major constituent of basement membrane (other components include laminin, nidogen, and heparan sulfate proteoglycan perlecan) and is a predominant type of collagen found in skeletal muscle. Other ECM genes affected by FGF2 treatment included laminins and fibronectins (Table 1). Most profoundly affected by FGF2 treatment were laminin gamma 1 (LAMC1) and laminin alpha 5 (LAMA5). qRT-PCR confirmed increased expression levels of these two laminins (Figure 4B). Fibronectin 1 was downregulated by FGF2 treatment (Figure 4B). FGF2 treatment of human fibroblasts modulates production of the ECM. The ECM composition of the FGF2-treated fibroblasts favors the pro-regenerative outcome in the wound site directly by affecting the balance between scar formation and tissue regeneration and potentially thorough changes in cell attachment to ECM, cell migration, and cell proliferation.

Cell attachment to the ECM is regulated through integrins, heterodimers that recognize specific substrates. Adhesion and migration on collagen substrate is performed through α1β1 and α2β1 and formation of collagen type I and type III network is dependent on fibronectin and α2β1 [25-27]. We show FGF2-induced upregulation of β1 and α2 (Figure 4B). Integrins α5β1, αVβ3, and α4β1 pairs are utilized to bind fibronectin matrix [28,29], αVβ5 is used to adhere to vitronectin, and α6β1, α2β1, α3β1 to adhere to laminin and entactin [10,12,30]. FGF2 treatment downregulated ITGB2 and upregulated ITGB3 and ITGA10 (Figure 4B). Integrins connect ECM to actin cytoskeleton via focal adhesions rich in talin, which is recruited to F-actin, and binds integrin pairs, which in turn leads to transmission of F-actin movements to ECM [29]. Change in the composition of integrins, as well as in the components of focal adhesions leads to change in migration, as well as preferential binding to specific substrate, production of which is regulated by FGF2 treatment, and may benefit a pro-regenerative response.

During wound healing, fibroblasts acquire a highly migratory phenotype. The process is driven by actin polymerization and resulting microfilaments of the cell’s leading edge link to ECM via integrins. Actomyosin contraction then allows for the disassembly of adhesions in the rear and movement forward [31]. Thus, movement of the fibroblasts in the wound site is regulated not only by the ECM and adhesion molecules, but also by the actin cytoskeleton. Actin cytoskeleton is also involved in fibroblast contractile phenotype. During dermis healing, fibroblasts generate stress fibers (weakly contractile actin bundles) to enable contraction [22]. Fibroblasts’ shape is regulated by the environment and cell-matrix adhesion determines the cell shape, such as strong cell-ECM adhesion promotes spindle-shaped fibroblast [32]. *In vitro* fibroblasts were shown to have different morphology depending on the substrate they are grown on; in 3D cultures resembling an *in vivo* environment, fibroblasts display elongated shape, well-developed actin cortex, and filopodia at the leading edge [33]. Alpha actin ACTC1, which is a constituent of the contractile apparatus, was downregulated in human dermal fibroblasts treated with FGF2 (Table 2; Figure 5B). Gamma actin ACTG2, which is involved in cellular motility and adhesion, was 64-fold downregulated (Table 2), though qRT-PCR did not confirm its expression. By regulating cytoskeleton gene expression, FGF2 potentially promotes cell migration in the wound site, and reduces contraction that leads to the favorable pro-regenerative outcome.

Previously, it was shown that administration of FGF2 alone into a dermal wound shows reduced scar formation [15], which can be attributed to upregulation of matrix metalloproteinase MMP1 [17]. Our data shows strong upregulation of MMP1 (Figure 4B), the metalloproteinase responsible for cleaving collagen type I, II, and III [34]. FGF2 signaling was shown to activate the MMP1 promoter [35]. MMP1 was able to improve the skeletal muscle regeneration process by reducing scar tissue formation [36-38] and by promoting migration of myoblasts involved in regeneration of skeletal muscle [39,40]. Interestingly, integrin α2β1 was shown to increase MMP1 expression [41,42]. By transplanting FGF2 treated human dermal fibroblasts, continuous increase in production of MMP1 among other factors, may be allowed, indicating that MMP1 is present not only at the time of the resolution phase of wound healing leading to decreased collagen production, but also at earlier stages of wound healing, for example during the inflammation stage. Other MMP molecules, such as stromelysins MMP3, MMP10, MMP11, were upregulated as well (Table 1 and Figure 4). MMP3 was previously shown to be responsible for contraction of fibroblasts during wound healing [43] and was regulated by FGF2 in a mouse model [44]. MMPs, mostly MMP2, 3, 9 and 10, are highly upregulated during amphibian limb regeneration [45-47]. All of these observations point toward a favorable role of MMPs in the regeneration process. Thus, FGF2-stimulated change in transcriptional profile of various MMPs is an important factor contributing to the regeneration-competence of fibroblasts.

FGF2 treatment also led to a favorable ratio between MMPs and tissue inhibitors of metalloproteinases (TIMPs), as an imbalance between MMPs and TIMPs has been shown to increase scar formation. FGF2 upregulated TIMP4 and downregulated TIMP3 expression (Figure 4B). ADAM and ADAMTS proteinases that were shown to be differentially regulated by FGF2 (Table 1) are regulators of ECM and adhesion molecules and affect cell motility, adhesion, and signaling during wound healing processes. ADAMTS1 and ADAMTS5 were downregulated by FGF2 treatment (Figure 4B). ADAM transmembrane proteinases are involved in cleaving and activating various cell surface molecules, whereas ADAMTS are secreted proteinases that can bind ECM. ADMATS8 that was upregulated by FGF2 treatment (Figure 4B) has anti-angiogenic properties [48].

The ratio of TGFB1/TGFB3 is a factor that predicts scar formation, the decrease in this ratio being indicative of reduced scar formation [11]. Fetal wounds that are known to heal without scar formation exhibit decreased TGFB1 levels [49]. Administration of TGFB3 has also been shown to reduce scar formation [15]. TGFB pathway was previously shown to be induced by FGF2 treatment in mouse embryonic fibroblasts (MEFs) [50]. We observed no change in the levels of TGFB1 due to FGF2 treatment by microarray analysis whereas qRT-PCR showed downregulation of TGFB1 levels (Figure 5B). We observed upregulation of TGFBR3 due to FGF2 treatment by the array, but qRT-PCR showed no change in expression levels (Figure 5B). qRT-PCR confirmed downregulation of TGFBR1 (Figure 5B). These observations may be due to differences between mouse embryonic fibroblasts and adult human dermal fibroblasts, indicating that FGF2 response in these cells may be unique.

Decreasing inflammation has been shown to decrease scar formation. For example, when wounds of skin and oral mucosa were compared, there was less inflammation and scarring in oral mucosa [51]. Non-scar wound healing in fetal wounds is also characterized by absence of inflammation [52-56]. Inflammatory events are integrated by chemokines. Chemokines are chemotactic cytokines that regulate migration of cells during inflammatory process. ELR(^+^) CXC chemokines are neutrophil attractants and activators. CXCL6 or granulocyte chemotactic protein-2 (GCP-2) is a ELR(^+^) CXC chemokine. FGF2 treatment led to increase in CXCL6 chemokine expression (Figure 5B). CXCL5, a chemokine that attracts and activates neutrophils, amplifies inflammatory cascade, and stimulates local production of cytokines was shown to be upregulated by FGF2 treatment (Figure 5B). Interestingly, when CXCL5 is cleaved by MMP1, 2, 8, 9, and 13, increased inflammation is observed and cell recruitment to the wound site is activated [57]. CCL2 (monocyte chemoattractant protein-1, MCP-1), which is involved in inflammatory cell recruitment, can be induced through focal adhesion kinase (FAK) leading to inflammation and scar production in a cutaneous injury, and CCL2 knock-out mice showed decreased scarring [58]. Here, we observed downregulation of CCL2 due to FGF2 treatment (Figure 5B). In agreement with previous publications, implantation of FGF2 treated fibroblasts, which show CCL2 downregulation, into a mouse wound sight leads to reduced scar formation [19]. We also show in this transcriptome analysis that IL6/STAT3 signaling pathway is regulated by FGF2 (Figure 5B). Interleukin 6 (IL6) is a pleiotropic cytokine that is produced by a variety of cells such as epidermal cells, endothelial cells, and fibroblasts [59]. IL6 is known to increase production of collagen [60], thus the decrease in collagen synthesis that we observe in skeletal muscle injury, can be partially explained by decrease in IL6. CCL2 was shown to induce IL6 secretion in human lung fibroblasts, and has a role in regulating fibrosis [61] and was shown previously to be regulated by FGF2 [62]. Scarless, fetal wounds are characterized by diminished expression of pro-inflammatory IL6 and IL8 [52,53]. Here, we show that FGF2 treatment significantly reduces IL6 levels (Figure 5B), whereas levels of IL8 are upregulated with FGF2 treatment (Table 3). FGF2-induced decrease in IL6 level could be contributing to pro-regenerative phenotype of adult human fibroblasts. Signal transducer and activator of transcription (STAT3) conveys signals from IL6. Loss of IL6 was shown to result in deficiency of proliferation and migration of myoblasts [63-65]. IL6/STAT3 was shown recently to be involved in excessive ECM production and increased cellular proliferation in hypertrophic scars compared to normal human fibroblasts [66].

## Conclusions

Comparison of transcriptomes between control and regeneration-competent fibroblasts indicates significant differences in expression of genes involved in several biological processes during wound healing. Downregulation of collagens, upregulation of ECM remodeling enzymes, and downregulation of pro-inflammatory cytokines may be in part responsible for the cells’ pro-regenerative phenotype. A choice between scar-forming and pro-regenerative wound healing responses may depend on a balance between ECM production, degradation, consequent ECM contractility, and decreased inflammatory response. Further studies are needed to elucidate functional significance of specific disregulated genes.

## Methods

### Cell culture

Adult human dermal fibroblasts were obtained from ATCC (CRL-2352) at passage number 1 (p1). Cells were expanded using culture conditions recommended by the supplier, namely ambient oxygen, 5% CO_2_ in air, 37°C in DMEM/F12 and 10% FBS. Expansion was done by trypsinizing (0.05% trypsin, Cellgro) the cells at 80% confluence and replating them at a density of 14,000 cells/cm^2^. Cells were cryopreserved with DMSO and the same passage used for all the experimental groups. Cells from the same passage number 7 were grown for seven days at 5% O_2_, 5% CO_2_, 37°C in DMEM/F12 and 10% FCIII in one of the following culture conditions: 1. with 4 ng/ml human recombinant FGF2 (PeproTech) on tissue culture plastic; 2. with 4 ng/ml human recombinant FGF2 on glass culture surface; 3. on tissue culture plastic; and 4. on glass culture surface. After 7 days, cells were used for three RNA collections for transcriptome arrays and qRT-PCR.

### RNA isolation

Total RNA was isolated from all treatment groups using TRIZOL reagent (Invitrogen) following manufacturer’s protocol.

### OneArray microarray sample and data processing

To obtain a sense of global effects of surface and FGF2, two independent samples (in three technical replicates each) of cells grown on glass, glass with FGF2, plastic, and plastic with FGF2 were hybridized to the Human Whole Genome OneArray® v5 (Phalanx Biotech, Palo Alto, CA). RNA quality and integrity were determined utilizing an Agilent 2100 Bioanalyzer (Agilent Technologies, Palo Alto, CA, USA) and absorbance at A260/A280. Only high quality RNA, having a RIN of >7.0, and an A260/280 absorbance ratio of >1.8, was utilized for further experimentation. RNA was converted to double-stranded cDNA and amplified using *in vitro* transcription that included amino-allyl UTP, and the aRNA product was subsequently conjugated with Cy5™ NHS ester (GEH Lifesciences). Fragmented aRNA was hybridized at 50°C overnight using the HybBag mixing system with 1X OneArray Hybridization Buffer (Phalanx Biotech), 0.01 mg/ml sheared salmon sperm DNA (Promega, Madison, WI, USA), at a concentration of 0.025 mg/ml labeled target. After hybridization, the arrays were washed according to the OneArray protocol. Raw intensity signals for each microarray were captured using a Molecular Dynamics™ Axon 4100A scanner, measured using GenePixPro™ Software, and stored in GPR format.

### Data analysis

The data was analyzed with R/bioconductor using standard statistical functions and analysis modules for the ANOVA, T test, FDR, and functional analysis [67,68]. Analysis was performed in the following order. First, data was background corrected, normalized, and filtered to remove probes with very low expression or low variance (expression but no variation) across conditions. Next, 2-way ANOVA was performed to determine significant gene probes for the two factors and possible interactions between cell culture surface and FGF2. LIMMA package was used to determine significantly differentially expressed genes (DEG) with moderate t-statistic as main statistic of significance and standard errors moderated using Bayesian model [69-71]. P-values were adjusted for multiple comparisons using Benjamini and Hochberg method to control the false discovery rate (FDR) [72]. FDR cutoff value of 0.05 was used.

Gene Ontology (GO) analysis was performed to analyze functional enrichment within DEG due to FGF2 treatment in human dermal fibroblasts cultured on plastic. DEG due to FGF2 treatment were profiled for GO slim using geneListPie package [73]. In order to perform GO analysis, GOstats package was used [74]. Hypergeometric conditional testing was performed to obtain overrepresented GO terms that belong to three groups: biological process, molecular function, and cellular component.

### Quantitative RT-PCR

cDNA was prepared from total RNA using QuantiTect Reverse Transcription kit (Qiagen) using mixture of oligo-dT and random primers method. The kit also includes elimination of genomic DNA step prior to reverse transcription. 1 μg of total RNA was used for cDNA preparation. For each qPCR reaction 20 ng of cDNA were used. qPCR was performed using SYBR SELECT master mix (Invitrogen). The list of primers can be found in Additional file 7. Quantification of qPCR results was performed by the ΔΔCT method.

### Availability of supporting data

The data sets supporting the results of this article are available in Gene Expression Omnibus (GEO) repository (GSE48967) and at http://users.wpi.edu/~tdominko/iRC_transcriptome/.

## Competing interests

The authors declare that they have no competing interests.

## Authors' contributions

All authors read and approved the final manuscript. OK and TD drafted manuscript. DLP, EFR, SA and OK performed the bioinformatics analysis. SA and OK harvested the cells. OK performed qRT-PCR analysis.

## Supplementary Material

Additional file 1 **11,124 filtered probes.** Matrix of background corrected, normalized, and filtered log2 intensity values. Click here for file

Additional file 2 **Pearson’s correlation coefficients.** Scatter plots and correlation coefficients comparing two biological replicates for each of four experimental groups: A. adult human dermal fibroblasts cultured on glass with addition of 4 ng/ml FGF2, B. adult human dermal fibroblasts cultured on glass, C. adult human dermal fibroblasts cultured on plastic with addition of 4 ng/ml FGF2, and C. adult human dermal fibroblasts cultured on plastic. Click here for file

Additional file 3 **Glass FvsU.** List of significantly differentially expressed genes due to FGF2 treatment of adult human dermal fibroblasts cultured on glass. Click here for file

Additional file 4 **Plastic FvsU.** List of significantly differentially expressed genes due to FGF2 treatment of adult human dermal fibroblasts cultured on tissue culture plastic. Click here for file

Additional file 5 **GOslim.** GO slim terms associated with biological process, molecular function, and cellular component of genes affected by FGF2 treatment of adult human dermal fibroblasts grown on tissue culture plastic. Click here for file

Additional file 6 **GOterms.** Results of functional annotation using Gene Ontology. GO terms belong to three groups: biological process, molecular function, and cellular component. Click here for file

Additional file 7 **Primers.** List of primers used for qRT-PCR analysis. Click here for file
