# Supplementary figures and images for: FGF2-induced effects on transcriptome associated with regeneration competence in adult human fibroblasts
Source: BMC Genomics. 2013 Sep 26;14:656. doi: 10.1186/1471-2164-14-656 (PMC3849719; doi:10.1186/1471-2164-14-656)

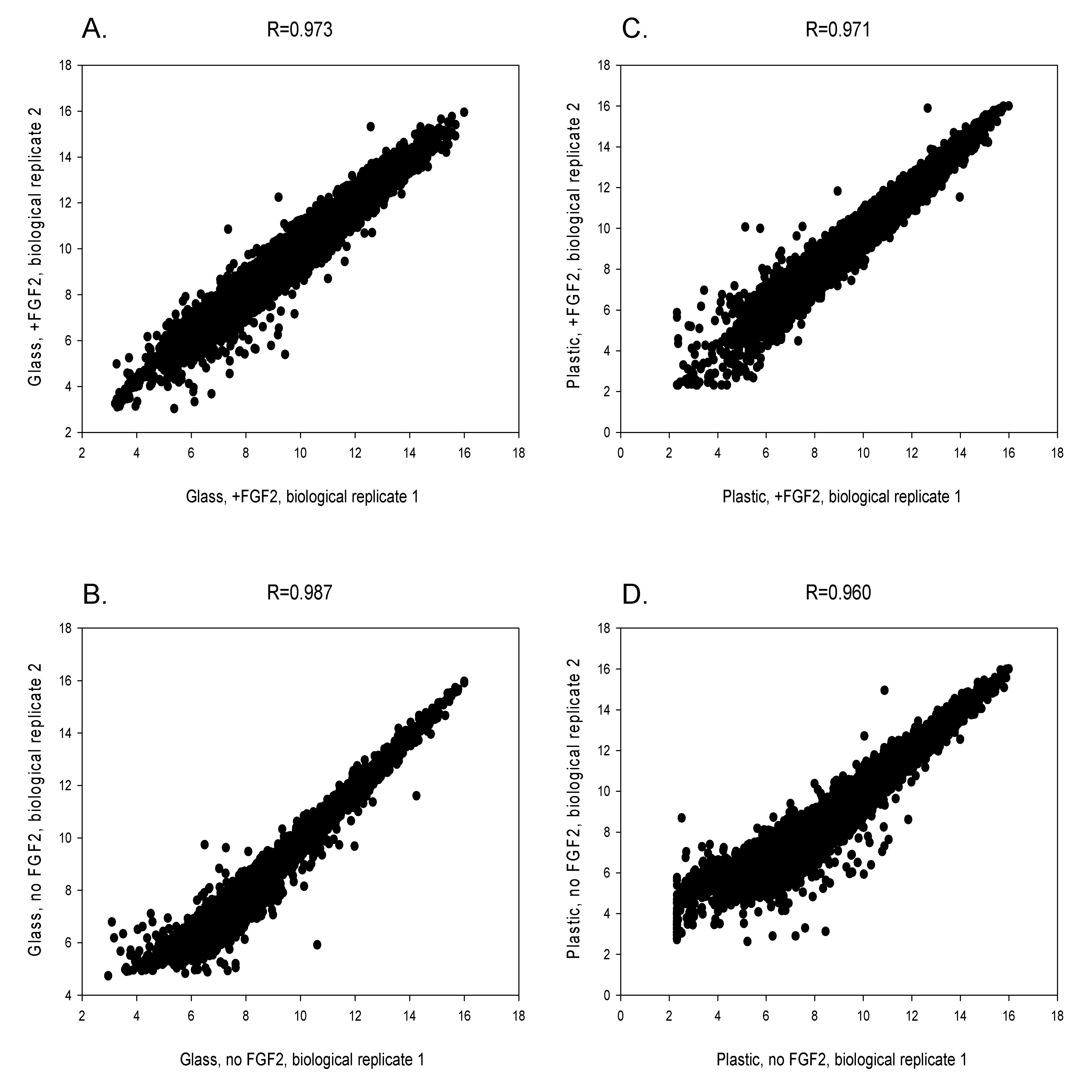

Supplement: Additional file 2 — Pearson’s correlation coefficients. Scatter plots and correlation coefficients comparing two biological replicates for each of four experimental groups: A. adult human dermal fibroblasts cultured on glass with addition of 4 ng/ml FGF2, B. adult human dermal fibroblasts cultured on glass, C. adult human dermal fibroblasts cultured on plastic with addition of 4 ng/ml FGF2, and C. adult human dermal fibroblasts cultured on plastic. [file 1471-2164-14-656-S2.png]
